# Global research trends, reporting and handling of missing data in observational studies of type 2 diabetes mellitus with mild cognitive impairment from 2020 to 2025: a systematic review

**DOI:** 10.3389/fendo.2026.1649881

**Published:** 2026-05-11

**Authors:** Yang Liu, Maoyi Yang, Yifan Liao, Zhipeng Hu

**Affiliations:** Hospital of Chengdu University of Traditional Chinese Medicine, Chengdu University of Traditional Chinese Medicine, Chengdu, China

**Keywords:** bibliometrics, mild cognitive impairment, missing data, observational study, systematic review, type 2 diabetes

## Abstract

**Background:**

Missing data is common in observational studies, and even more so in type 2 diabetes mellitus with mild cognitive impairment(T2DM-MCI), which limits the completion of assessments. We evaluated the extent, current reporting, and handling of missing data, as well as the prevailing research trends in observational studies related to T2DM-MCI.

**Methods:**

A systematic search of PubMed, Embase, and Cochrane Library was conducted from January 2020 to April 2025 to identify observational studies related to T2DM-MCI. Bibliometrics was performed using VOSviewer and CiteSpace to evaluate publishing trends, authors, journals, and keywords. The reporting and handling of missing data were assessed according to the guidelines recommended by STROBE and Sterne et al., with a focus on the recording, causes, mechanisms, processing methods, and sensitivity analysis of missing data. Data analysis was conducted using SPSS 26, and visualization was performed using Origin Pro 2024.

**Results:**

Among the 4,471 screened records, 88 studies (78 in English and 10 in Chinese) were included in this analysis. Among the 78 English articles, the annual publication volume exhibited fluctuations, peaking in 2024. Chinese institutions and authors led in research output. *Diabetes, Metabolic Syndrome, and Obesity* had the highest publication volume (7, 8.97%). Keyword identified five clusters: 1) resting-state functional magnetic resonance imaging, 2) metabolic disorders, 3) clinical assessment tools, 4) molecular mechanisms, and 5) emerging fields such as the gut microbiome.

**Missing data:**

Only 22.7% (n = 20) of the studies quantified the missing data, with an average of 9.1%. Among studies with missing data (n = 23), 52.2% (n = 12) provided reasons for missing data, primarily citing poor quality of data collection (41.7%) and loss to follow-up (41.7%). Complete case analysis was the predominant method for addressing missing data (93.3%). No study articulated the hypothesized mechanisms underlying the missing data, and only 4.4% (n = 1) performed a sensitivity analysis.

**Conclusion:**

In the domain of T2DM-MCI, research outcomes post-COVID-19 pandemic indicate a rebound, with China maintaining a leading position in scientific research output. However, the reporting of missing data remains ambiguous, and the methods employed to handle such data are insufficient, which may potentially introduce bias.

**Systematic Review Registration:**

https://doi.org/10.17605/OSF.IO/EZDXM.

## Introduction

Diabetes Mellitus is a severe chronic disease that significantly impacts individuals, families, and society. In 2019, the global prevalence of diabetes was estimated at 9.3% (463 million people), with projections indicating an increase to 10.9% (700 million people) by 2045 (1). As a highly prevalent metabolic disorder, type 2 diabetes mellitus (T2DM) is recognized as a critical risk factor for cognitive dysfunction (2, 3). Large-scale epidemiological studies have demonstrated that the risk of dementia in patients with T2DM is significantly higher than in non-diabetic populations. The underlying pathological mechanisms involve cumulative damage to the central nervous system via multiple pathways, including chronic hyperglycemia, insulin resistance, vascular damage, and neuroinflammation (4, 5).

In the spectrum of cognitive impairments associated with diabetes, mild cognitive impairment (MCI) occupies a significant position. Specifically, type 2 diabetes mellitus with mild cognitive impairment (T2DM-MCI) refers to the clinical condition wherein patients with T2DM experience cognitive decline that exceeds the expected age-related changes, affecting domains such as memory, executive function, attention, or language. However, these patients do not meet the diagnostic criteria for dementia, and their fundamental daily living abilities remain intact (6). It is noteworthy that while MCI is regarded as an intermediate stage between a normal cognitive state and dementia, individuals with MCI face an elevated risk of progressing to dementia (7). Thus, early identification and intervention for MCI are of paramount importance.

In recent years, numerous observational studies have focused on exploring the association between T2DM and MCI, analyzing risk factors, pathological features, and potential biomarkers. These studies provide a crucial basis for clinical risk stratification and prevention strategies. However, they often encounter significant methodological challenges due to inherent selection or recall biases among participants and the suboptimal quality of data collection, particularly concerning missing data. A review of complex, large-scale epidemiological surveys indicates that inadequate reporting and improper handling of missing data remain prevalent (8–14). The results from such studies can introduce substantial selection bias, diminish statistical power, and ultimately distort the true strength and direction of the association between T2DM and MCI (15–17).

It is noteworthy that, despite the considerable impact of missing data on the reliability of conclusions, current research on the models, mechanisms, and processing methods for missing data in observational studies of T2DM-MCI remains underdeveloped. Most existing articles focus on the simplistic application of single statistical methods (18–20) and lack in-depth discussion on systematic evaluation and strategies for handling missing data within the T2DM-MCI domain. Therefore, systematically evaluating the characteristics of missing data in observational studies of T2DM-MCI and elucidating its potential impact mechanisms are essential for enhancing research quality in this area and ensuring the validity of conclusions.

This study aims to address this gap by reviewing the reporting and handling of missing data in observational studies of T2DM-MCI across multiple databases from 2020 to 2025. Furthermore, it seeks to identify current research hotspots through bibliometric and visual analysis, thereby providing a foundation and guidance for the standardized design and data analysis of high-quality observational studies in the future.

## Methods

### Data sources and search strategy

We conducted a comprehensive search of PubMed, Embase, and Cochrane Library for studies publishedbetween January 1, 2020, and April 24, 2025, to evaluate the globalization trends and the status of missing data processing in observational studies on T2DM-MCI. The search strategy was developed with assistance from the Harvard University Library and experienced medical professionals, incorporating key search terms such as cohort study, cross-sectional study, case-control study, type 2 diabetes, and cognitive impairment. Due to the limitations in reviewing a substantial number of identified records, we restricted our search to publications from 2020 to 2025. The detailed search strategy is provided in **Supplementary Material 1**.

### Study selection

Abstracts of the identified citations were screened for eligibility based on the following criteria: (i) observational studies (cohort studies, cross-sectional studies, case-control studies); (ii) subjects diagnosed with T2DM-MCI. We excluded meta-analyses, randomized controlled trials, animal studies, research protocols, and guidelines. Summaries of meetings were also excluded as they did not provide sufficient information regarding the handling of missing data. Two researchers independently conducted preliminary screenings of titles and abstracts, as well as full-text re-screening. Any disputes were resolved through consultation with the third and fourth researchers.

### Data extraction and analysis

We extracted the following components for our analysis: research identity (including title, author, keywords, publication year, and journal), research setting, research design (prospective, retrospective, or cross-sectional), data collection methods, sample size, primary statistical analysis methods, and information regarding missing data. The handling of missing data adheres to the guidelines recommended by STROBE and Sterne et al. (21, 22). Specifically, we applied the following items from the STROBE statement that are directly relevant to missing data: Item 12c (explain how missing data were addressed), Item 12e (describe any sensitivity analyses), Item 13a (report numbers of individuals at each stage of the study, e.g., numbers potentially eligible, examined for eligibility, confirmed eligible, included in the study, completing follow-up, and analyzed), Item 13b (give reasons for non-participation at each stage), Item 14b (indicate the number of participants with missing data for each variable of interest), and Item 17 (report other analyses done, e.g., analyses of subgroups and interactions, and sensitivity analyses).

To ensure transparency and reproducibility, we predefined a standardized coding scheme for data extraction. The extent of missing data was classified as “quantified” (explicit number or percentage reported), “not reported”, or “unclear”. Reasons for missingness were categorized as “poor data collection quality”, “loss to follow-up”, or “other”. Missing data mechanisms were assessed based on the assumptions stated in each study (Missing Completely At Random [MCAR], Missing At Random [MAR], Missing Not At Random [MNAR], or not stated). Handling methods were classified as “complete case analysis”, “multiple imputation”, or “other”. Sensitivity analysis was recorded as “yes” (with description) or “no”. Two researchers independently extracted all data; disagreements were resolved through discussion or consultation with the third and fourth researchers.

Consistent with this framework, we documented the number of missing data points, their causes, the mechanisms behind them, the methods employed to address them, and whether a sensitivity analysis was conducted. In addition, we followed the reporting framework proposed by Sterne et al. for multiple imputation. In instances of multiple imputations, we recorded the variables utilized, the number of imputations performed, the assessment of the imputation process, and the treatment of non-normal or categorical variables. For all variables with incomplete observations, we opted to select the missing covariates or the highest values of the outcome data to avoid redundant calculations. If the extent of missing data for the primary outcome was not clearly stated, we calculated this by determining the difference between the number of participants included in the analysis and the number of registered participants. Based on the reporting and handling of missing data, the data were summarized as frequencies and proportions and analyzed using IBM SPSS Statistics 26.

### Bibliometrics and visualization analysis

This study employs Origin Pro 2024 software to analyze trends and proportions of annual publications. Additionally, the data extracted was analyzed and visualized using CiteSpace (version 5.7.R5, 64-bit) and VOSviewer (version 1.6.20). VOSviewer, developed by Waltman et al. in 2009, is a free, Java-based software designed for analyzing large volumes of literature data and presenting it in a map format (23). In this study, VOSviewer is utilized to generate visual charts that identify the highest-yielding journals, authors, and high-frequency keywords. To visualize research results in specific areas through the construction of a literature co-citation network, Professor Chen Chaomei developed CiteSpace (version 5.7.R5), which employs experimental frameworks to explore new concepts and evaluate existing technologies (24). This facilitates a deeper understanding of knowledge domains, research frontiers, and trends, while also aiding in the prediction of future research trajectories. This study leverages CiteSpace to visualize keyword clustering and author clustering.

## Results

### Literature retrieval and characteristics

**Figure 1:**
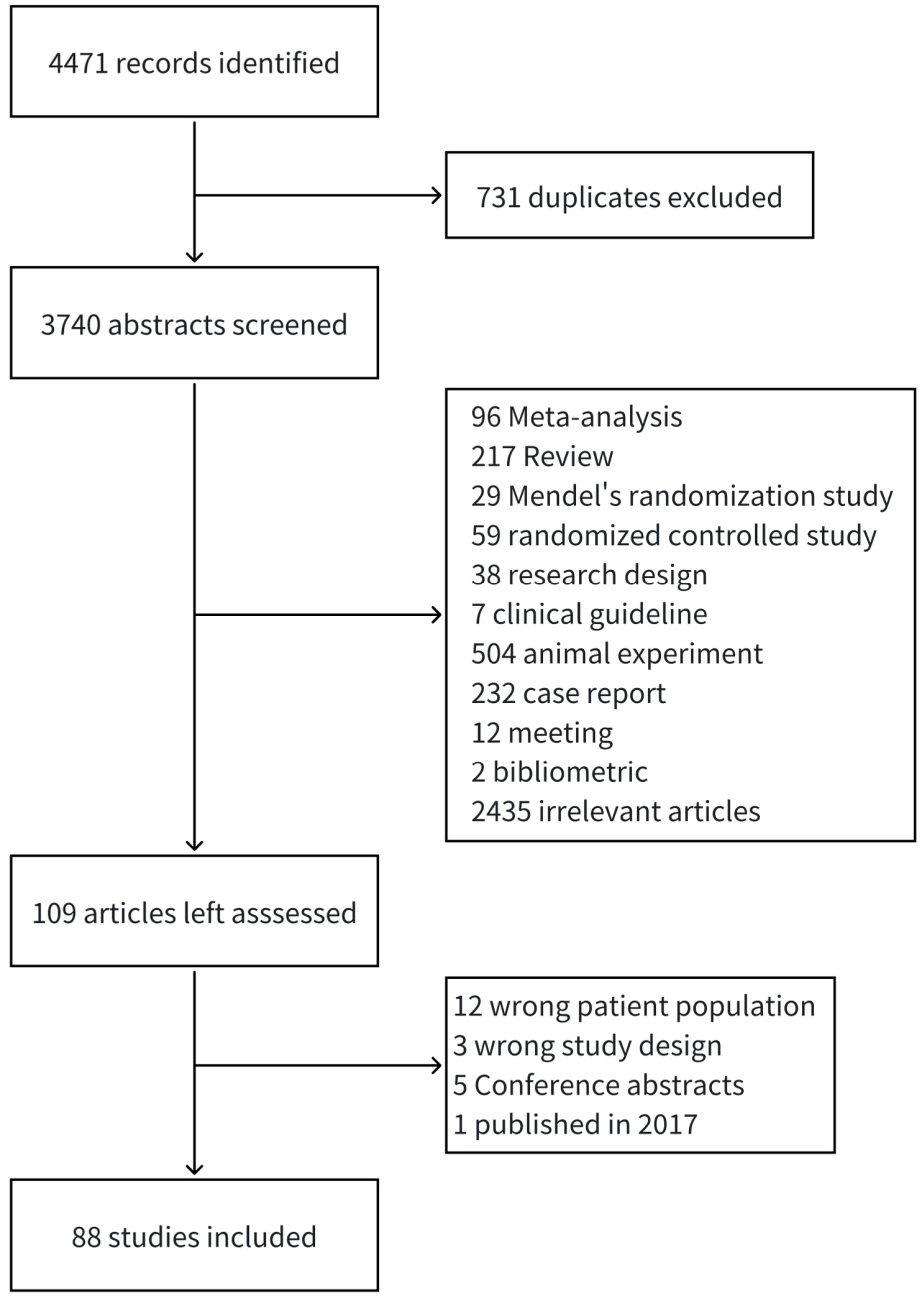
Flowchart of study inclusion process.

**Figure 2:**
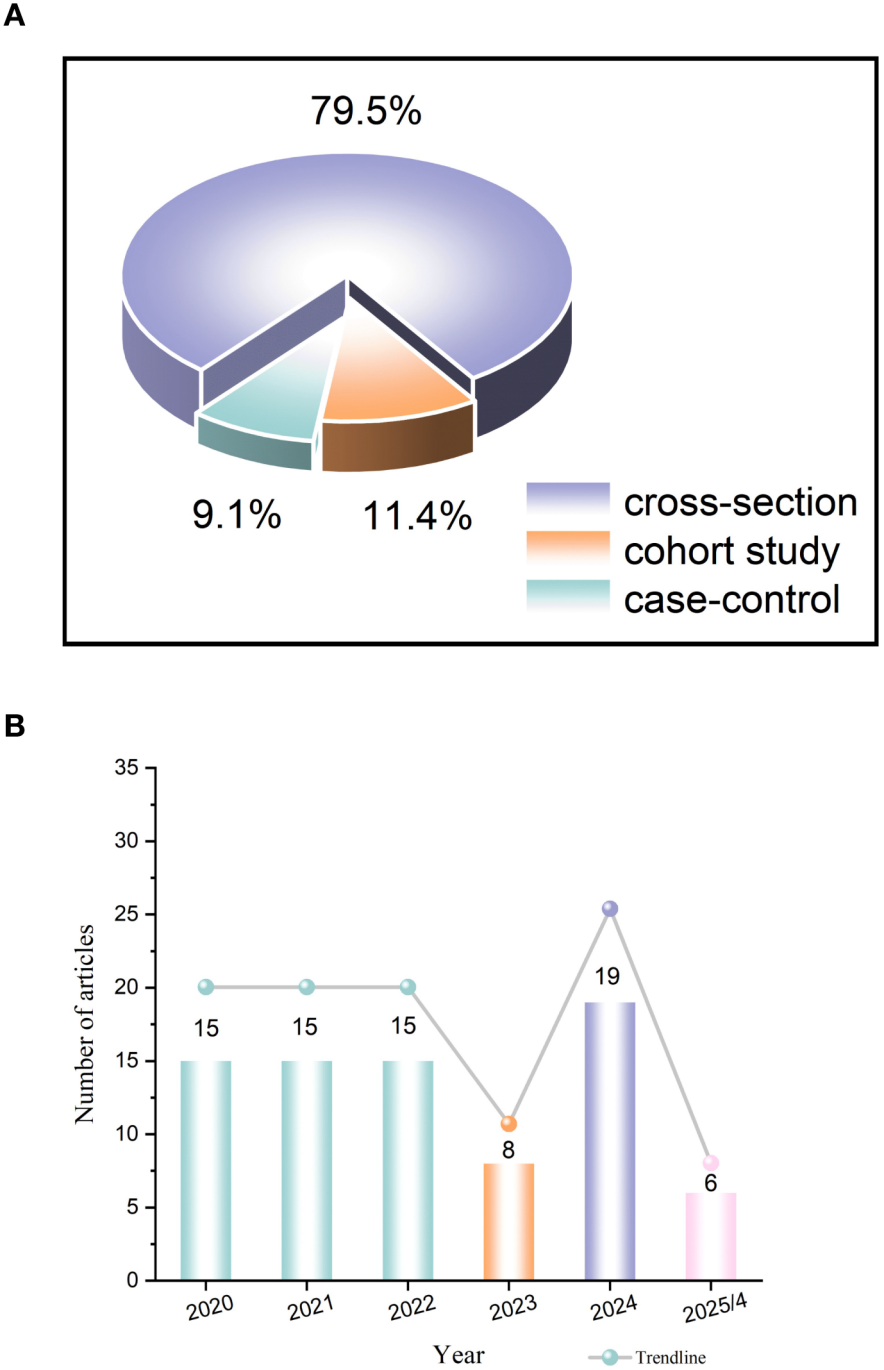
**(A)** Proportion of study types included. **(B)** A Trend chart of annual publication volume.

**Figure 1** illustrates the flowchart of the included studies. A total of 4,471 articles were retrieved, from which 731 duplicate articles were removed, leaving 3,740 abstracts for screening. Among these, 3,631 articles that did not meet the inclusion criteria were excluded. This exclusion comprised 96 meta-analyses, 217 reviews, 29 Mendelian randomized studies, 59 randomized controlled trials, 38 research programs, 7 clinical guidelines, 504 animal experiments, 232 reports, 12 conference proceedings, 2 bibliometric studies, and 2,435 unrelated articles. The remaining 109 articles were read in full text, and 21 were further excluded. Ultimately, a total of 88 studies were included in the review, consisting of 78 articles in English and 10 articles in Chinese. The included studies primarily employed a cross-sectional design (70,79.5%), with single-center studies accounting for 97.7% (n = 86). Data were predominantly collected through questionnaire surveys (72,83.3%). The median sample size was 194 (IQR: 108-321), with 75.0% of the studies having a sample size ranging from 100 to 1,000 cases (**Table 1**; **Figure 2A**).

**Table 1:** Characteristics of included studies.

Description	Total (n=88)
Study design, n (%)	
Prospective	7 (7.9)
Retrospective	11 (12.5)
Cross-Section	70 (79.6)
Sample size, Median (IQR)	194 (108-321)
Sample size, n (%)	
<100	17 (19.3)
100-1000	66 (75.0)
>1000	5 (5.7)
Study site, n (%)	
Multisite	2 (2.3)
Single site	86 (97.7)
Method of data collection, n (%)	
Administrative data	11 (12.5)
Surveys^a^	72 (83.3)
Mixed	5 (5.7)

n, number;%, percent.

^a^ includes clinical report form or any study questionnaire.

We conducted a visual analysis of the final English literature, which encompassed 78 articles from 37 countries and regions, published in 49 journals by 236 institutions and authored by 504 individuals. Since 2020, the annual number of publications has generally shown an unstable trend (**Figure 2B**). We categorize the annual volume of publications into three distinct stages. From 2020 to 2022, during the rampant spread of COVID-19, the number of publications stabilized at 15 per year, indicating sustained interest from researchers in this field. The publication rate remained relatively stable during this period. In 2023, as the COVID-19 epidemic came under control and people’s lives gradually returned to normal, the number of published articles decreased to 8. This decline suggests that, in the first year following the control of the epidemic, researchers showed reduced interest in this area of study. However, by April 2025, the number of publications had increased, peaking in 2024. This trend indicates that the domain has garnered increased attention since 2024.

### Authors and journals

**Figure 3:**
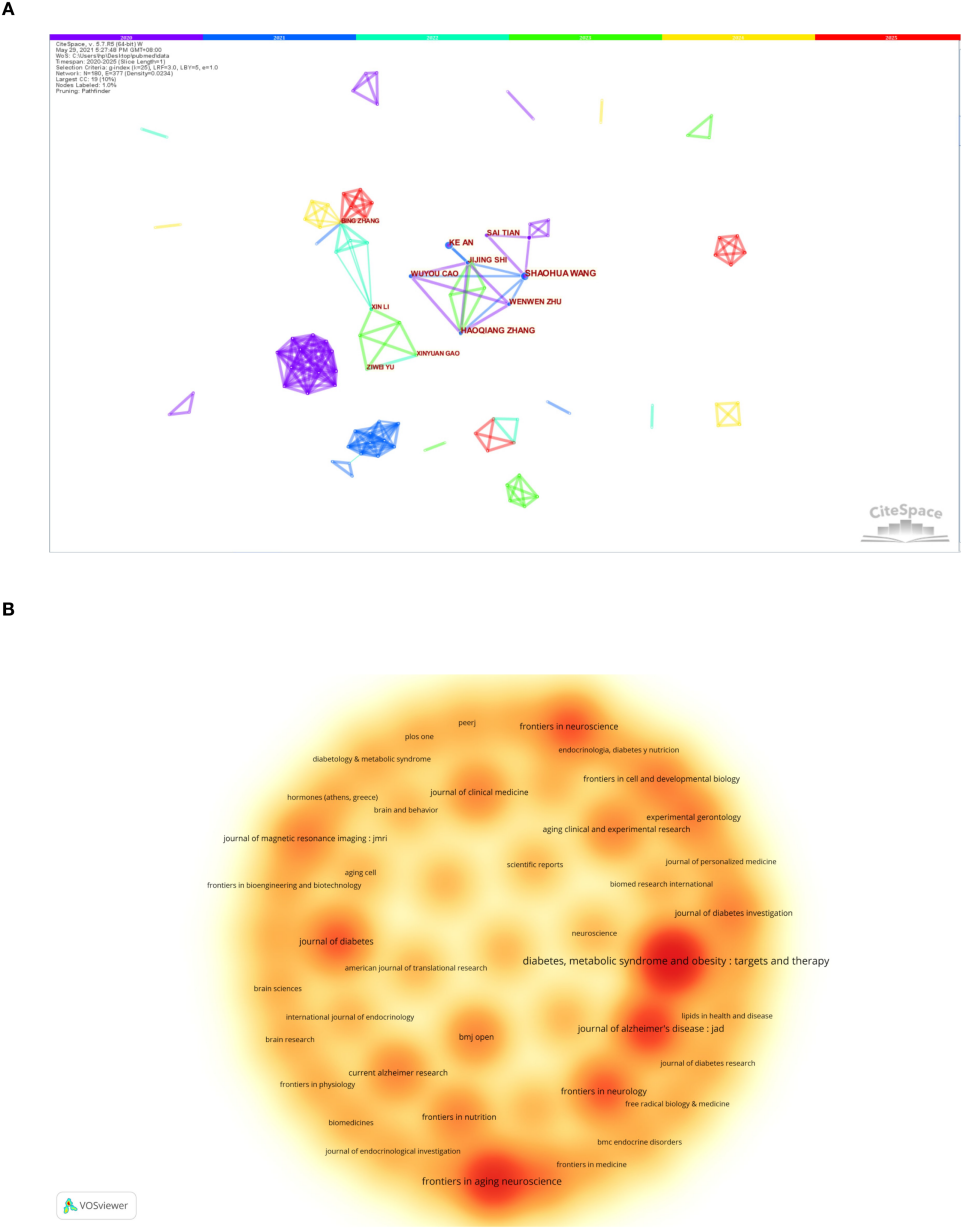
**(A)** A Collaboration network diagram among authors. **(B)** Density chart of published literature in journals.

A total of 504 authors conducted observational studies on T2DM-MCI. **Table 2** presents the top 10 authors who have published in the past five years, all of whom are from China, including Affiliated Zhongda Hospital of Southeast University, First Affiliated Hospital of Harbin Medical University, and Nanjing Drum Tower Hospital Clinical College of Nanjing Medical University. Among them, the earliest publication dates of the top seven authors were all in 2020, suggesting that they may have begun to concentrate on this field earlier than the last three authors. The collaboration network among the authors (**Figure 3A**) indicates that ShaoHua Wang, the author with the highest output, maintains a close cooperative relationship with the six authors following him. All of these authors are affiliated with the Affiliated Zhongda Hospital of Southeast University, and their publication dates are primarily concentrated between 2020 and 2023. Bing Zhang and Xin Li also exhibit a strong collaboration, being affiliated with Nanjing Drum Tower Hospital. Most of their publications occurred in 2022, indicating that this group began to focus on this field relatively later. Further analysis of the cooperation network indicates that collaboration within institutions is robust, while inter-agency cooperation remains limited. Consequently, we advocate for enhanced collaboration among authors from different institutions to expand the sample size of observational studies and provide more scientifically robust and convincing evidence for the findings.

**Table 2:** Statistics of literature published in the top 10 authors.

Rank	Author	Articles counts	Year
1	Shaohua Wang	9	2020
2	Haoqiang Zhang	7	2020
3	Ke An	7	2020
4	Sai Tian	6	2020
5	JiJing Shi	6	2020
6	Wenwen Zhu	6	2020
7	Wuyou Cao	6	2020
8	Xin Li	5	2022
9	Ziwei Yu	4	2022
10	Bing Zhang	4	2021

**Table 3**; **Figure 3B** present the ten most productive journals in the field. The journal with the highest number of published papers is Diabetes, Metabolic Syndrome and Obesity (7 papers, 8.97%), followed by Frontiers in Aging Neuroscience (5 papers, 6.41%), Journal of Alzheimer’s Disease (4 papers, 5.13%), and Journal of Diabetes (3 papers, 3.85%). Among these top ten journals, Frontiers in Aging Neuroscience has the highest impact factor (IF) of 4.1. All listed journals are categorized as Q1, Q2, or Q3, with the Journal of Magnetic Resonance Imaging classified as Q1.

**Table 3:** Statistics of literature published in the top 10 journals.

Rank	Journal	Articles counts	Percentage (78)	IF	Quartile in category
1	Diabetes, Metabolic Syndrome, and Obesity	7	8.97	2.8	Q3
2	Frontiers in Aging Neuroscience	5	6.41	4.1	Q2
3	Journal of Alzheimer’s Disease	4	5.13	3.4	Q2
4	Journal of Diabetes	3	3.85	3	Q2
5	Frontiers in Neuroscience	3	3.85	3.2	Q2
6	Frontiers in Neurology	3	3.85	2.7	Q2
7	Journal of Magnetic Resonance Imaging	2	2.56	3.1	Q1
8	Journal of Diabetes Investigation	2	2.56	3.1	Q2
9	Journal of Clinical Medicine	2	2.56	3	Q1
10	Frontiers in Nutrition	2	2.56	4	Q2

### Keywords

**Figure 4:**
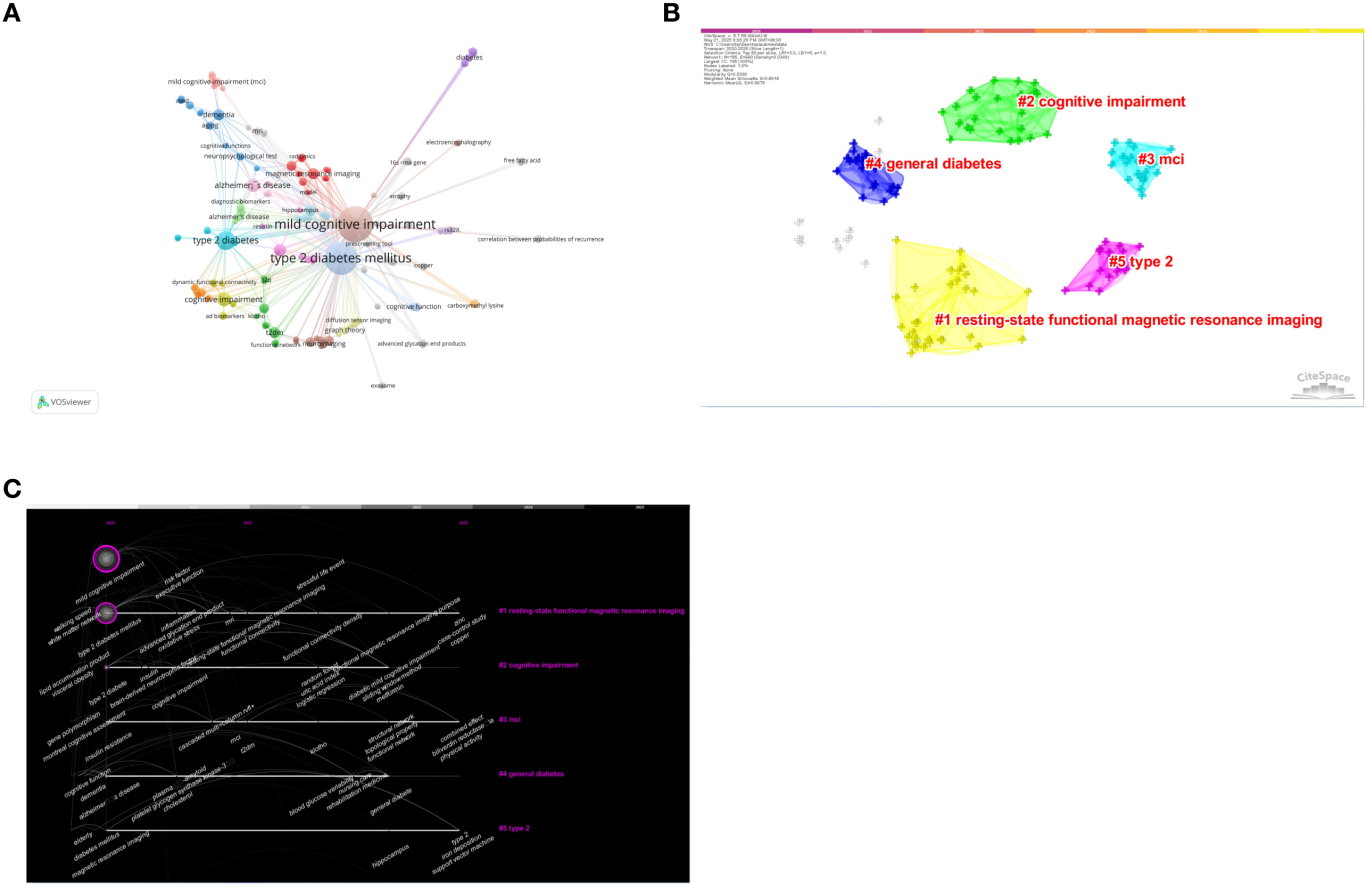
**(A)** High-frequency keywords network diagram. **(B)** Cluster analysis of keywords. **(C)** Keywords clustering timeline plot.

Through the analysis of keywords, we can gain a comprehensive understanding of the general situation and developmental direction of this field. Utilizing the co-occurrence of keywords in VOSviewer software, we identified that, in addition to T2DM and MCI, the most frequently occurring keywords are Alzheimer’s disease (8), followed by insulin resistance (5), dementia (4), and magnetic resonance imaging (4) (**Table 4**; **Figure 4A**). We clustered the keywords and constructed a network comprising 119 keywords, resulting in a total of five distinct clusters (**Figure 4B**). Cluster 1 (yellow) contains 30 keywords, including functional connectivity network and white matter network, which are associated with neuroimaging markers. Cluster 2 (green) comprises 27 keywords, such as insulin resistance, blood glucose fluctuation, lipid abnormality, and uric acid index, which pertain to various metabolic disorders. Cluster 3 (blue) includes 23 keywords, such as the Montreal Cognitive Assessment (MoCA) and neuropsychological tests, primarily serving as clinical assessment tools. Cluster 4 (dark blue) consists of 22 keywords, including oxidative stress, brain-derived neurotrophic factor (BDNF), and other molecular mechanisms. Finally, Cluster 5 (red) contains 17 keywords, primarily focusing on the intestinal microbiome, galectin-3, and other emerging directions. We utilized CiteSpace software to create a clustering map that visualizes the evolution of research hotspots over time (**Figure 4C**).

**Table 4:** List of high-frequency keywords.

Rank	Keyword	Counts	Rank	Keyword	Counts
1	mild cognitive impairment	57	11	proteomics	2
2	type 2 diabetes	56	12	executive function	2
3	Alzheimer’s disease	8	13	Montreal cognitive assessment	2
4	insulin resistance	5	14	brain-derived neurotrophic factor	2
5	dementia	4	15	elderly	2
6	magnetic resonance imaging	4	16	insulin	2
7	neuropsychological test	4	17	white matter network	2
8	functional connectivity	3	18	functional connectivity density	2
9	resting-state functional magnetic resonance imaging	3	19	neuroimaging	2
10	aging	2	20	cerebral small vessel disease	2

### Burst analysis of keywords

**Figure 5:**
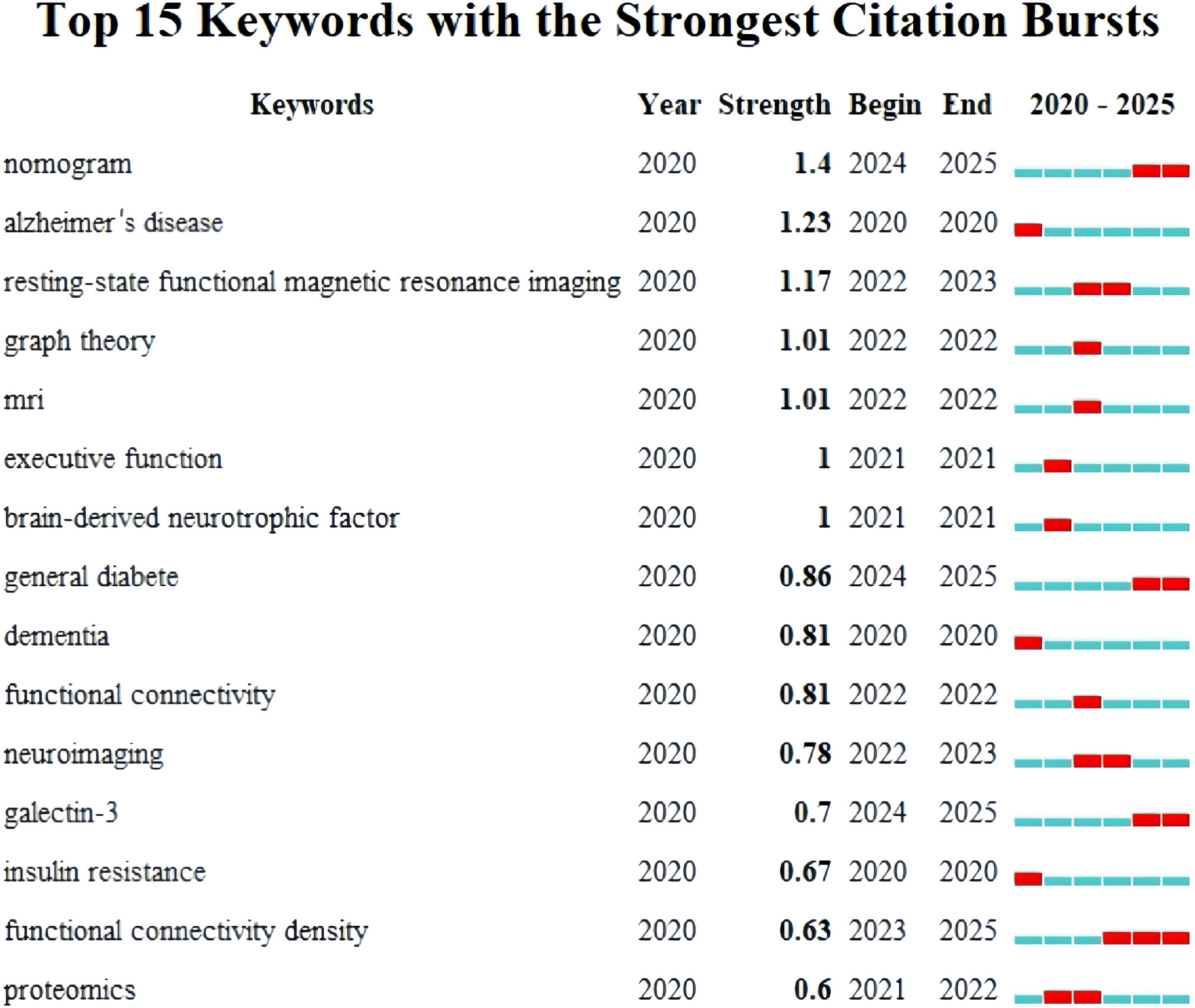
Visualization chart of keywords with the strongest citation bursts.

We focused on 15 keywords that exhibited the strongest outbreaks in this field (**Figure 5**), including Alzheimer’s disease, resting-state functional magnetic resonance imaging, executive function, insulin resistance, dementia, and brain-derived neurotrophic factor. Notably, nomogram, galectin-3, and functional connectivity density have garnered particular attention over the past two years. These keywords signify the current research hotspots and potential future research trends in this area.

### Report of missing data

To clarify the relationship between the numbers of studies reported in **Tables 5**, **6**, we define the following sub-sets: among all 88 included studies, 23 studies (26.1%) explicitly acknowledged the presence of missing data (either by quantifying it or by stating that some data were missing). Within these 23 studies, 20 (87.0%) reported the exact amount of missing data (mean 9.1%, range 1.23–38.1%), while the remaining 3 studies acknowledged missing data but did not quantify the amount, and 15 studies (65.2%) described a specific method for handling missing data. Notably, among the 20 studies that quantified missing data, 5 (25.0%) did not proceed to describe any analytical strategy for addressing those missing values, indicating a gap between recognition of missing data and appropriate methodological handling. The remaining 65 studies (73.9%) did not mention missing data at all. Among the 23 studies that acknowledged missing data, 14 (60.9%) excluded participants based on missing data. Of these 14 studies, 13 (92.9%) explicitly reported the number of individuals excluded, while 1 study did not provide this information.

**Table 5:** Reporting of missing data.

Description	n (%)
Reported the amount of missing data (N = 88)	
Yes	20 (22.7)
No	65 (73.9)
Unclear	3 (3.4)
Reported reasons for missing data (N = 23)	
Yes	12 (52.2)
No	11 (47.8)
Reported number of individuals excluded due to missing data (N = 14)	
Yes	13 (92.9)
No	1 (7.1)
Described method used to handle missing data (N = 23)	
Yes	15 (65.2)
No	8 (34.8)
Stated the assumptions for missing data methods (N = 15)	
Yes	0 (0)
No	15 (100)

Of the 88 included studies, 20 (22.7%) reported the amount of missing data, 65 (73.9%) did not, and 3 (3.4%) acknowledged missing data but did not quantify it. Among the 23 studies with missing data (including the 20 that quantified and the 3 that did not), 12 (52.2%) gave reasons for missingness; among the 14 studies that excluded participants due to missing data, 13 (92.9%) reported the number excluded; among the 23 studies, 15 (65.2%) described a handling method; and among those 15, none stated the assumed missing data mechanism (MCAR, MAR, or MNAR).

**Table 6:** Handling of missing data.

Description	n (%)
Methods used for dealing with missing data (N = 15)	
Complete case analysis	14 (93.3)
Multiple imputation	1 (6.7)
Compared differences between individuals with and without incomplete data (N = 14)	
Yes	0 (0.0)
No	14 (100.0)
Performed sensitivity analysis to test robustness of results (N = 23)	
Yes	1 (4.4)
No	22 (95.6)
**For multiple imputation (N = 1)**	
Indicated number of imputed datasets	
No	
Reported variables included in imputation model	
No	
Described handling of non-normal and categorical variables	
Yes	
Evaluated multiple imputation analysis	
No	

Of the 15 studies that handled missing data, 14 (93.3%) used complete case analysis and 1 (6.7%) used multiple imputation. Among the 14 studies that excluded cases, none compared those with versus without missing data. Only 1 of the 23 studies with missing data (4.4%) performed a sensitivity analysis. The single study using multiple imputation did not report the number of imputations, imputation variables, or model evaluation, but did describe handling of non-normal and categorical variables.

“For multiple imputation (N = 1) separately evaluates the reporting quality of the only study that employed multiple imputation, based on the guidelines recommended by Sterne et al. This study did not indicate the number of imputed datasets, did not report the variables included in the imputation model, and did not evaluate the multiple imputation analysis; however, it did describe the handling of non‑normal and categorical variables.”

The report detailing the missing data is presented in **Table 5**. A total of 77.3% (n = 68) of the studies either did not mention missing data or provided ambiguous information regarding its extent, while only 22.7% (n = 20) quantified the proportion of missing data (mean 9.1%, range 1.23-38.1%). Additionally, 52.2% (n = 12) of the studies offered explanations for the missing data, primarily attributing it to poor record quality (41.7%) and loss to follow-up (41.7%). Most studies (92.9%) reported the number of exclusions due to missing data. Among the 23 studies that documented missing data, the majority (15, 65.2%) described methods for addressing the issue. Unfortunately, none of the studies specified the types of missing mechanisms assumed in their analyses.

### Processing of missing data

**Table 6** presents the methods employed to address missing data in the included studies. Among the studies that reported their strategies for handling missing data (n = 15), complete case analysis emerged as the most prevalent method, with approximately 93.3% of the studies (n = 14) excluding individual observations of missing data as part of their inclusion criteria, either at the initial stage or during the analysis phase. In the 14 studies that omitted participants based on data integrity, no comparisons were made between missing and non-missing data. Only 4.4% (n = 1) of the studies assessed the robustness of their missing data handling regarding the results. Notably, only one study employed the Multiple Imputation method for missing data processing, which did not adhere to the Sterne guidelines. This study only reported its approach to addressing non-normal distributions and categorical variables, without detailing the number of imputed datasets, the variables included in the imputation model, or the evaluation of the multiple imputation analysis.

## Discussion

This study systematically analyzes the global research patterns of T2DM-MCI from 2020 to 2025 using bibliometric methods for the first time. The results indicate that, despite fluctuations in the annual number of publications in this field due to the impact of COVID-19, research interest has significantly rebounded since 2024. This trend suggests an increasing attention from the academic community toward the association between T2DM and cognitive impairment. Chinese scholars hold a dominant position in this field, with high-yield authors and institutions primarily concentrated in the Affiliated Zhongda Hospital of Southeast University. This concentration may be attributed to China’s large population and its status as having the highest number of diabetic patients globally (25). Furthermore, it reflects China’s active contribution to the interdisciplinary study of metabolic and neurodegenerative diseases. However, both international and domestic collaboration networks remain limited, highlighting the need for strengthened cross-agency cooperation to enhance the diversity and universality of research.

Keywords can reflect the research frontiers and trends in a field. We conducted cluster analysis, co-occurrence analysis, and burst analysis on keywords. In general, there are three core directions in the current research field:(i) the application of neuroimaging techniques, such as resting-state functional magnetic resonance imaging, in the early diagnosis of MCI;(ii) the interaction between metabolic disorders, such as insulin resistance and lipid abnormalities, and neuroinflammation; and (iii) the potential effects of the gut microbiome and genetic polymorphisms on cognitive function. Notably, the burst trend of emerging keywords, such as functional connectivity density and galectin-3, suggests that future research may focus on exploring molecular mechanisms and conducting multi-omics integrated analyses.

Overall, there is a significant deficiency in the reporting and handling of missing data in observational studies concerning T2DM-MCI. Common practices include inadequate and ambiguous reporting, the direct exclusion of participants with missing data, and a failure to evaluate the robustness of findings derived from missing data. These issues are also evident in other fields and various studies (26–29).

In some of the included studies, it was not specified whether the data were missing or fully observed, a common oversight in many contemporary reports (30–35). The absence of explanations for missing data can mislead readers and undermine the critical assessment and reproducibility of research outcomes. The average proportion of missing data reported in studies was 9.1%, and improper handling of this data can introduce significant bias (36–40).

We compared them with a previous methodological review by Karahalios et al. (2012), which examined missing data in cohort studies with repeated exposure measures (26). That study reported that 43% of articles reported the amount of missing data, and 66% used complete case analysis. Our findings show even lower reporting rates (only 22.7% quantified missing data) and a much higher reliance on complete case analysis (93.3%), suggesting that the handling of missing data in T2DM-MCI observational studies may be even more problematic than in general cohort studies a decade ago. However, unlike earlier reviews that largely focused on longitudinal designs, our study reveals that even in predominantly cross-sectional designs (79.6% of included studies), the handling of missing data remains suboptimal. This persistence of poor reporting across different study designs underscores a widespread methodological gap that has not improved over the past decade. The predominance of cross-sectional designs (79.6%) in our included studies has important implications for missing data patterns and handling strategies. In cross-sectional studies, missing data typically arise from incomplete questionnaires or failed measurements at a single time point, often driven by participant refusal, item non-response, or administrative errors (14). By contrast, longitudinal studies face additional challenges such as attrition and intermittent missingness over time, which may require more sophisticated methods like mixed-effects models or multiple imputation with time-varying covariates (21). Despite these differences, we observed that even in cross-sectional studies, the vast majority (93.3%) resorted to complete case analysis – a method that assumes missingness is Missing Completely At Random (MCAR). However, in cross-sectional health surveys, missingness is often related to observed characteristics (e.g., older age, lower education, or poorer health status), violating MCAR and potentially biasing results. Therefore, the reliance on complete case analysis in cross-sectional T2DM-MCI studies is particularly concerning. Future research should adopt more robust methods, such as multiple imputation under the Missing At Random (MAR) assumption.

A notable gap identified in our review is the disconnect between acknowledging missing data and providing an analytical solution. Among the 20 studies that quantified missing data, only 15 (75%) described a method for handling it. This means that 5 studies (25% of those with quantified missing data) explicitly reported that data were missing but offered no information on how those missing values were addressed in the statistical analysis. This omission is critical because readers cannot determine whether the reported results are based on a reduced sample (complete case analysis) or some form of imputation. The absence of handling methods, even when missing data are recognized, represents a missed opportunity to enhance transparency and reproducibility. It also increases the risk of selective reporting bias, as authors may implicitly adopt complete case analysis without stating it, thereby understating the potential impact of missing data on their conclusions. Therefore, we urge authors to adhere to the STROBE checklist.

In the included studies, complete case analysis was excessively reliant (93.3%) on data processing, while alternative methods, such as multiple imputation, were infrequently employed. This trend aligns with previous studies (41–45), indicating a low adherence to existing guidelines, such as STROBE, in practice. The application of complete case analysis is predicated on the assumption that the missing data isMCAR, implying that the absence of data is unrelated to both observed and unobserved variables (46). Consequently, the fully observed sample is expected to represent the overall study population adequately. However, the validity of this assumption diminishes as the proportion of missing data increases; significant missing data not only reduces statistical efficiency but also heightens the risk of distortion due to selection bias (47). Interestingly, some studies exclude participants with missing data based on data integrity criteria established during the initial inclusion phase, thereby creating a ‘complete observation’ dataset. Although such practices mitigate the issue of missing data, they may inadvertently introduce biases if systematic differences exist between the complete data group and the group with missing data (48, 49). Unfortunately, the studies included in our analysis did not assess whether differences existed between these two groups when employing complete case analysis. Furthermore, regardless of the missing data mechanism or methodology utilized, the robustness of test results against various alternative hypotheses and methods serves as one of the critical means of evaluating bias (22, 46). However, we observed that sensitivity analyses were conducted in only a limited number of studies (1.1%).

Multiple imputation is a commonly used statistical technique for dealing with missing values in data sets. Based on the MAR assumption, this assumption is considered to be valid in many longitudinal data environments (50). Based on the missing mechanism of existing data and assumptions, it generates possible values to replace missing observations to reproduce multiple complete versions of the original data set, and then combines them into a single result. And the values filled in between multiple data sets are different, reflecting the uncertainty of missing values (51). Unlike complete case analysis, this approach utilizes all available data, thereby minimizing the loss of accuracy and statistical power (21, 52). However, existing guidelines for multiple imputations recommend that researchers clearly describe the procedural elements to facilitate subsequent reviews (21); unfortunately, these details are often inadequately reported in studies. Among the studies reviewed, only one employed multiple imputation methods, and it failed to fully disclose the selected variables, imputation variables, and details regarding model evaluation.

Overall, observational studies on T2DM-MCI exhibit a high proportion of missing data (average 9.1%), along with vague reporting and reliance on a singular processing method.

## Limitations

Through the use of visual analysis tools such as CiteSpace and VOSviewer, we have gained insights into the research progress and global trends in the field of T2DM-MCI. However, this study does have several limitations. Firstly, all reports included at the end of this review are observational studies, and their limited number may not adequately reflect the overall trends in the research field. Secondly, due to the existence of synonyms, abbreviations, and full names, keyword clustering may differ from the actual results. Therefore, the interpretation of these results should be approached with caution. Additionally, we did not exclude studies from the same participant cohort, which may lead to duplication or overlap of data and reports. Lastly, some studies are challenging to assess fully due to the vague methodological descriptions, which can obscure the impact of missing data. Future research should aim to expand the database and the coverage of years. Nevertheless, we hope that the data gaps highlighted in this review provide a comprehensive mapping of the current situation in the entire field.

## Conclusion

In observational studies concerning T2DM-MCI, the global research output has exhibited fluctuations at various stages, with a notable rebound since 2024. China leads in research volume; however, there is a lack of cross-agency and international collaboration. The primary journals contributing to core output include *Metabolic Syndrome and Obesity* and various *Frontiers* series. Currently, trending research directions encompass neuroimaging, metabolic disorders, molecular mechanisms, functional connectivity density, and galectin-3, among others. Concurrently, issues such as inadequate reporting and imprecise handling of missing data are prevalent in this domain. Many studies either fail to address missing data or exclude a significant number of participants with missing information. The methods employed for managing missing data tend to be relatively simplistic and non-standardized, with a majority of studies neglecting to assess the robustness of their results, thereby significantly heightening the risk of bias.

## Data Availability

The datasets presented in this study can be found in online repositories. The names of the repository/repositories and accession number(s) can be found in the article/**Supplementary Material**.

## Supplementary material

Supplementary Material 1 Search strategies for databases
